# Highly Accurate Step Counting at Various Walking States Using Low-Cost Inertial Measurement Unit Support Indoor Positioning System

**DOI:** 10.3390/s18103186

**Published:** 2018-09-20

**Authors:** Van Thanh Pham, Duc Anh Nguyen, Nhu Dinh Dang, Hong Hai Pham, Van An Tran, Kumbesan Sandrasegaran, Duc-Tan Tran

**Affiliations:** 1 Department of Electronics and Telecommunication, VNU University of Engineering and Technology, Hanoi 123000, Vietnam; 2 Department of Automation and Technical Equipment of Fire Fighting & Rescue, The University of Fire Fighting & Prevention, Hanoi 123000, Vietnam; anhpchp@gmail.com (D.A.N.); nhudinht34@gmail.com (N.D.D.); honghaipham.bk@gmail.com (H.H.P.); antv79@gmail.com (V.A.T.); 3 Department of Engineering and Information Technology, University of Technology Sydney, Sydney 2007, Australia; Kumbesan.Sandrasegaran@uts.edu.au

**Keywords:** Accelerometer, indoor positioning, low-cost IMU, smartwatch, step counting, various walking, smartphone

## Abstract

Accurate step counting is essential for indoor positioning, health monitoring systems, and other indoor positioning services. There are several publications and commercial applications in step counting. Nevertheless, over-counting, under-counting, and false walking problems are still encountered in these methods. In this paper, we propose to develop a highly accurate step counting method to solve these limitations by proposing four features: Minimal peak distance, minimal peak prominence, dynamic thresholding, and vibration elimination, and these features are adaptive with the user’s states. Our proposed features are combined with periodicity and similarity features to solve false walking problem. The proposed method shows a significant improvement of 99.42% and 96.47% of the average of accuracy in free walking and false walking problems, respectively, on our datasets. Furthermore, our proposed method also achieves the average accuracy of 97.04% on public datasets and better accuracy in comparison with three commercial step counting applications: Pedometer and Weight Loss Coach installed on Lenovo P780, Health apps in iPhone 5s (iOS 10.3.3), and S-health in Samsung Galaxy S5 (Android 6.01).

## 1. Introduction

The Global Positioning System (GPS) is unreliable for indoor localization applications. Therefore, an indoor positioning system is essential and attractive for both researchers and companies since it is employed in widespread practical applications; thus, several techniques have been proposed for it. Firstly, indoor positioning is based on pre-installed sensors/devices with a high accuracy of such as camera, wireless sensor network, wireless network, UWB (Ultra-wideband), and Doppler radar [1,2,3,4,5], but limitations of these techniques are that it is expensive and only applicable on pre-installed environments. Other methods use an accelerometer or IMU (Inertial Measurement Unit) that do not require a pre-installed support system, so it is suitable for unknown environments. However, it is shown that accuracy is not high. Hence, highly accurate step counting is an essential part to enhance the accuracy of an indoor positioning system in the case of using IMU. 

Many researches focus on developing step counting, such as the step counting algorithm in References [6,7,8,9,10,11,12,13,14,15,16,17]. The publications in References [6,7] are based on a built-in smartphone accelerometer and propose features for step counting. In Reference [6], the authors propose thresholds to detect the maximum peaks and minimum valleys followed by the use of the minimum distance feature (which is the minimal samples or time to appear a next peak or valley) to avoid the over-counting problem (which means that the number of steps detected is greater than that of steps executed). Nevertheless, the signal recorded from low-cost IMU contains noises. Hence, the proposed method in Reference [6] is not strong enough to solve over-counting and false walking (which means that a user in still state moves their hands or turns on the device, and pushes it to trousers pocket; the vibration of his leg or trousers) problems in low-cost IMU. The step counting method in Reference [7] proposed three new features: Periodicity (which means that the time or samples differ between the two neighboring peaks), similarity (which means that the difference between neighboring peaks for the left steps or right steps), and continuity (which means that the data recorded from user is smooth and continuous) to solve false walking. However, this algorithm still has a false negative error (the factor to determine if a step has been executed, but the device cannot detect it) in intermittent motion, while true peaks are eliminated in the case of false peaks detected in the noise signals. Therefore, under-counting (which means that the number of steps detected is smaller than that of step execution) and false peak detection (which means that the false steps are confirmed as true steps) problems are still encountered when applying these features in low-cost IMU. Furthermore, our proposed system is used to transfer data between the inside and outside of a building in fire conditions in which a pre-installed transmitter may be destroyed by flame or high temperature [16,17,18,19,20,21]. Both of these methods, which use a built-in smartphone accelerometer, are difficult to integrate other sensors and wireless modules to transfer data between the inside and outside of a building because the wireless module in smartphones cannot transfer signals between the inside and outside of a building if we do not install pre-installed transmitter. Furthermore, the performance of the system is unstable and depends on the type of accelerometer.

The publication in Reference [8] proposed two features for step counting. Firstly, the authors used FFT (fast Fourier transform) to recognize walking state depending on the walking frequency feature. Then, based on the walking duration feature, walking frequency was multiplied by walking duration to count the number of steps. This method did not depend on axes, but it is computationally expensive and does not focus on counting steps in fast walking or eliminates false walking.

The publication in Reference [9] used PAA (Piecewise Aggregate Approximation) and SAX (Symbolic Aggregate approximation) for estimating the number of steps in slow walking. This research mainly depended on the proposed algorithm to compare the accuracy of three commercial accelerometers in step counting at slow walking.

In Reference [10], the authors proposed to use low-cost MEMS sensors in a navigation system to combine peak detection with band-pass filter. Based on classifier results, the threshold values are different among the states of moving (walking, running, up stairs, and down stairs). This method uses SVM (Support Vector Machine) in state classification that will require a huge number of computations.

Another popular trend in step counting is the use of a wristband because it is convenient for users to count steps in health monitors and indoor positioning systems. There are a variety of researches that focus on developing the algorithms and devices, but the performance of these algorithms/devices is not stable because of noises, arm swings, and complex and unknown environments [12,14,22]. The publications in References [12,14] have evaluated step counting on the wristband. The publication in Reference [12] proposed dynamic thresholding for step counting in conditions of slow and intermittent walking. Nevertheless, this method still shows over-counting problem caused by noise signals and a false walking state. The publication in Reference [14] evaluated the performance of seven popular wristband brands and the result indicated that the performance of these devices is unstable in different wristband brands and activities.

Having analyzed the above limitations, this paper proposes to develop a highly accurate step counting method. This paper comprises four sections focusing on analyzing our proposed features and achieved results in comparison with other methods in publications and commercial applications. Section 1 is the literature review of several methods and their limitations. In Section 2, we analyze our proposed features (minimal peak distance, minimal peak prominence, dynamic thresholding, and vibration elimination) and combine them with peak detection, periodicity, and similarity features in Reference [7] to solve under-counting, over-counting, and false walking problems. Furthermore, our proposed method is based on the results of motion classification [23,24] in order to propose the most suitable threshold of each feature for each state. Section 3 has results and discussions, in which we analyze the achieved results and compare our proposed method with others and some commercial applications. Section 4 is the conclusion that summarizes the achieved results and the future works.

## 2. Materials and Methods

Our proposed method combined peak detection with suitable minimal peak distance (the minimal number of samples or time to appear next peak), minimal peak prominence (the horizontal line from current peak will extend both to the left and right of the peak until it crosses the higher peak or reaches the left or right of signal in a window), dynamic thresholding (the average of maximal and minimal values of *Acc*(*j*) data in a window size), and vibration elimination (using Root Mean Square (RMS) to compute the amplitude of acceleration to eliminate all peaks that fluctuated around gravity acceleration (g = 9.81 m/s^2^)) in step counting. 

The minimal peak distance will increase in slow walking to avoid over-counting and decrease in fast walking to avoid under-counting; minimal peak prominence and dynamic thresholding focus on eliminating noises and false peaks in signals; vibration elimination is used to eliminate vibration in signals. Then, periodicity and similarity features are used to solve abnormal and false walking problems. 

**Figure 1:**
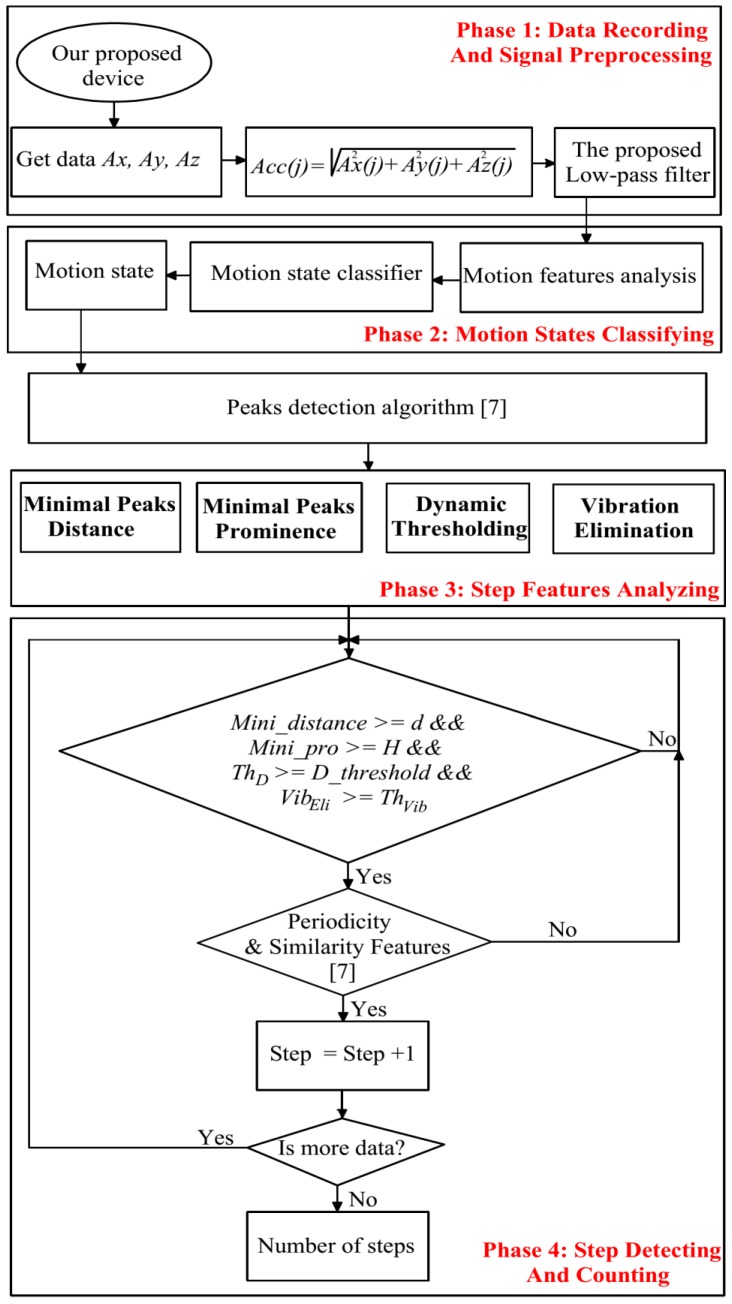
The flowchart depicting our step counting method.

In this paper, a 3-DOF accelerometer is used in our proposed method for step counting. Before applying our proposed features in step counting; the calibration, modeling, and simple Kalman filters are used to eliminate 3-DOF accelerometer errors. The detail of the calibration, modeling, and simple Kalman filter can be found in our prior research [25]. Then, 3-DOF accelerometer is used to record acceleration in three axes *Ax*, *Ay*, and *Az*. The proposed method is divided into four phases, as shown in Figure 1. In Phase 1, we record data and propose a suitable low-pass filter for preprocessing to eliminate noise in the received signal. In Phase 2, the proposed features in Reference [26] are used for motion state classification. In Phase 3, we propose four features for peak detection and Phase 4 presents conditions to check and count the number of peaks. If a peak satisfies all of the conditions in Phase 4, it will be confirmed as a step. The detail of each phase can be found in the following section:

Phase 1: Data recording and signal processing. The proposed system uses a 3-DOF accelerometer to record acceleration. Then, the Root Mean Square (RMS) of acceleration in three axes *Ax*, *Ay*, and *Az* is computed to reduce the dependence on orientation of the axis by using the following formula:

(1) $$Acc(j)=\sqrt{{\left(Ax(j)\right)}^{2}+{\left(Ay(j)\right)}^{2}+{\left(Az(j)\right)}^{2}}\;$$

where *Acc*(*j*) is the RMS of acceleration along three axes *Ax*(*j*), *Ay*(*j*), and *Az*(*j*) at time *j*; *Ax*(*j*) is the acceleration value in *Ax* axis at time *j*; *Ay*(*j*) is the acceleration value in *Ay* axis at time *j*; *Az*(*j*) is the acceleration value in *Az* axis at time *j*. In our proposed system, the sampling rate (*Fs*) of the sensor is 50 Hz. Normally, the step frequency is lower than 3 Hz (3 steps per second) [6,27] in all walking states (fast walking, normal walking and slow walking) [27]. To eliminate noise in the signal, we proposed to use a low-pass filter with a cutoff frequency = 3 Hz.

**Figure 2:**
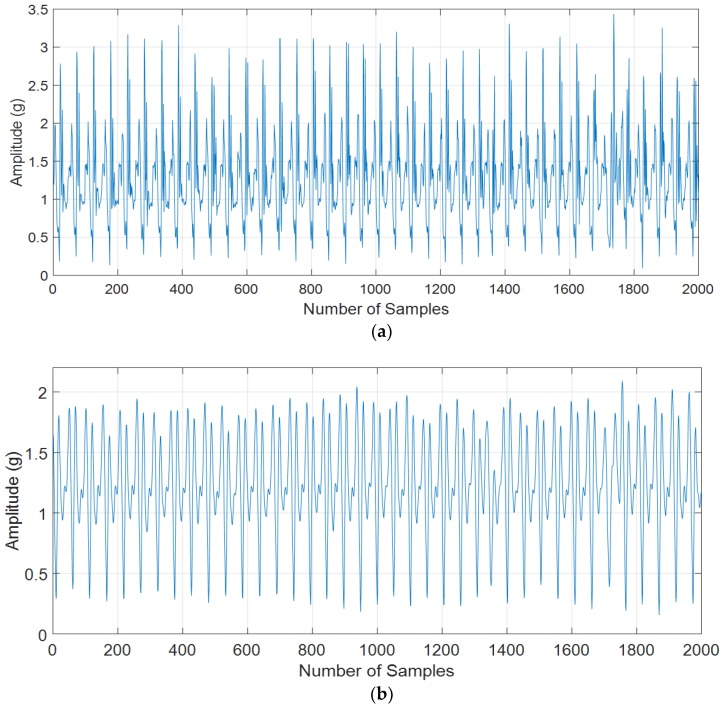
The signal before and after using the low-pass filter: (**a**) The raw signal before using the low-pass filter; and (**b**) The signal after using the low-pass filter.

The Figure 2 illustrates the signal before and after using our proposed low-pass filter. There are multi-false peaks in the signal without using low-pass filter (see Figure 2a) and these false peaks will be the cause of the over-counting problem. After using a low-pass filter, the signal is smoother and insignificant parts of the signal are eliminated (see Figure 2b).

**Figure 3:**
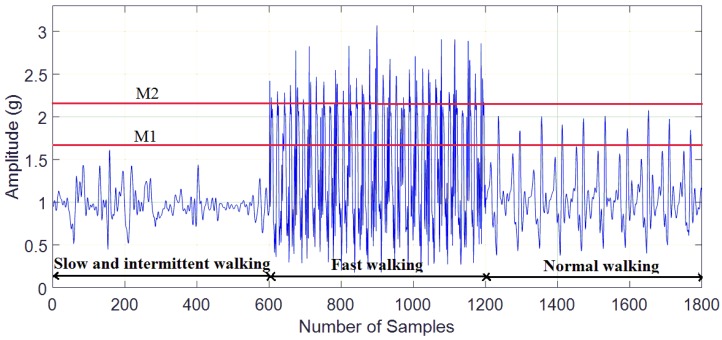
Acceleration magnitude of three different kinds of walking.

Phase 2: Motion states classifying. To distinguish among slow, normal and fast walking, we use the mean of the acceleration magnitude feature [23] to classify the motion state of walking. The feature is simple, but it effectively distinguishes the states of walking (see Figure 3).

It can be seen that the magnitude of fast walking is greater than that of normal walking and the amplitude of normal walking is greater than that of slow and intermittent walking. Hence, the thresholds M1 and M2 are proposed to distinguish among fast walking, normal walking, and slow and intermittent walking.

Phase 3: Step feature analyzing: Peak detection is used in variety of step counters with good performance. Nevertheless, the number of steps counted will depend on the minimal distance between two peaks. If the minimal peak distance is large, the counter will skip steps while a small value may cause over-counting. Hence, step counting based on peak detection is only suitable for true walking with a fixed state. If a person moves in a variety of states or false walking, peak detection-based step counting cannot solve the problem. Step counting based on periodicity, similarity and the continuity method [7] can solve the false walking problem, but it is the cause of true peak elimination because of noise when using 3-DOF low-cost sensors (see Figure 11b). In this paper, we propose four constraints: Minimal peak distance, minimal peak prominence, dynamic thresholding, and vibration elimination in peak detection in 3-DOF low-cost sensors. Then periodicity and similarity features are employed to solve false walking problem and enhance the reliable performance of our proposed algorithm.

● Minimal Peak Distance: Minimal peak distance is utilized to remove all peaks within “Minimal peak distance”. In our proposed system, the threshold of the minimal peak distance feature will depend on the motion states, which is greater in slow walking and smaller in fast walking. The suitable minimal peak distance is necessary since, if this value is large, it will ignore true peaks while small value is the cause of the over-counting problem [7]:

(2) $$Peak_{j}=Acc(j)\geq (Acc(j-d):Acc(j-1))\&\&Acc(j)\geq (Acc(j+1):Acc(j+d)),$$

where *Acc*(*j*) is detected peak at *j* position; and *d* is the minimal number of samples in both left and right sides of *Acc*(*j*). The value of d depends on the motion states. Based on the experimental results, we propose *d* = 12 for normal walking; *d* = 14 for slow walking; and *d* = 10 for fast walking state in this paper.

**Figure 4:**
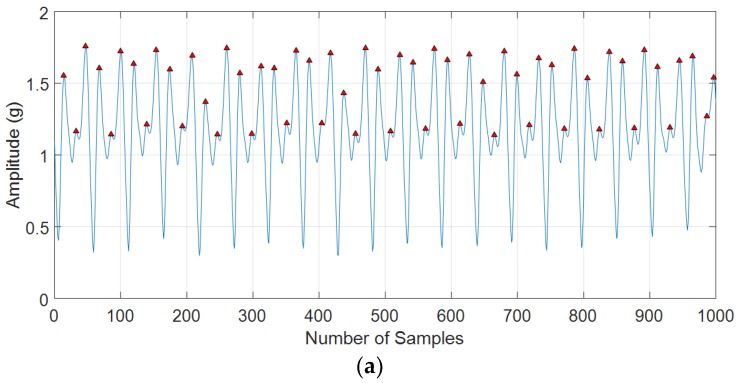

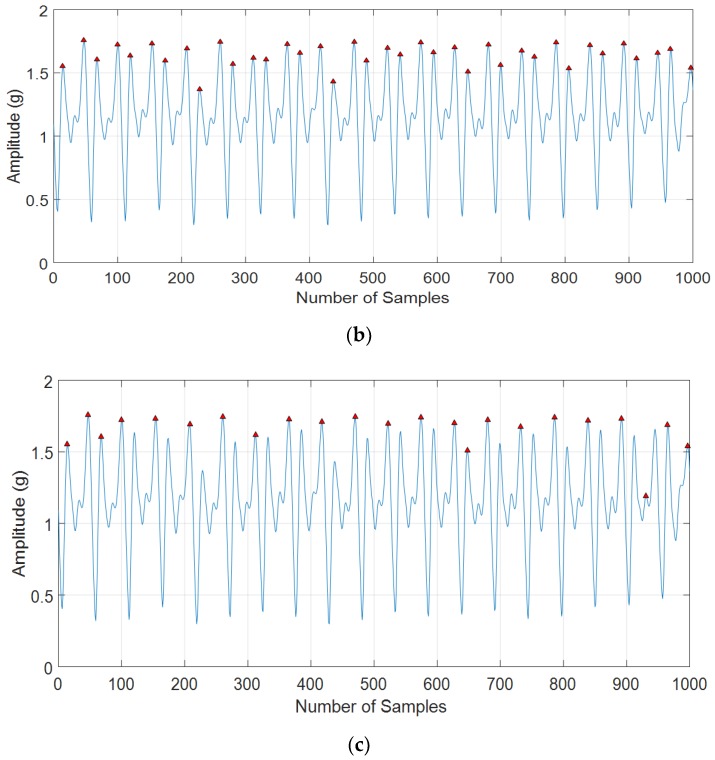
The *d* value on the peak detection in our normal walking data: (**a**) *d* = 5; (**b**) *d* = 14; (**c**) *d* = 20.

When the minimal peak distance is small (*d* = 5 in Figure 4a), there is a lot of false peak detection. Nevertheless, the large d value will be the cause of true peak elimination (*d* = 20 in Figure 4c). The algorithm is based on peak detection and a hard minimal peak distance threshold is not strong enough in step counting because the hard minimal peak distance threshold may be suitable with normal walking, but it is greater than the expected threshold value of fast walking and smaller than the expected threshold value of slow walking. The changing minimal peak distance is required for each moving state.

● Minimal peak prominence: This feature is a powerful technique that is used to remove the false peak by measuring the intrinsic height of the current peak, together with other peaks, by using a horizontal line from the current peak. The horizontal line will extend both to the left and right of the peak until it satisfied either of the following conditions [28,29]:

○ Crosses the higher peak
○ Reaches the left or right of the signal in a window

**Figure 5:**
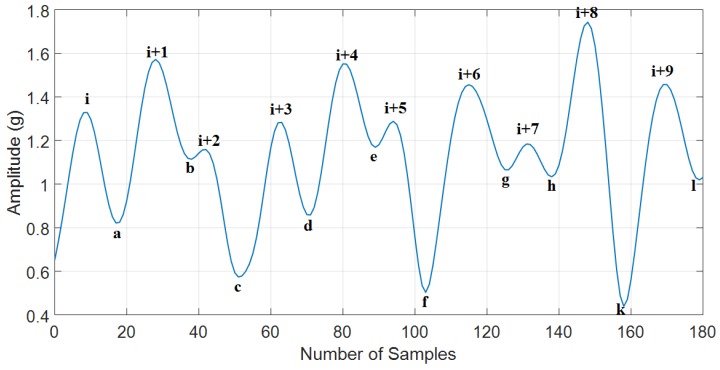
The minimal peak prominence definition.

The minimal peak prominence is either a signal valley or one of the signal endpoints in a window. The Figure 5 is an example to analyze the minimal peak prominence of the peaks (i), (i + 1), (i + 2), (i + 3), (i + 4), (i + 5), (i + 6), (i + 7), (i + 8), and (i + 9), and Table 1 has the details of the analysis process.

**Figure 6:**
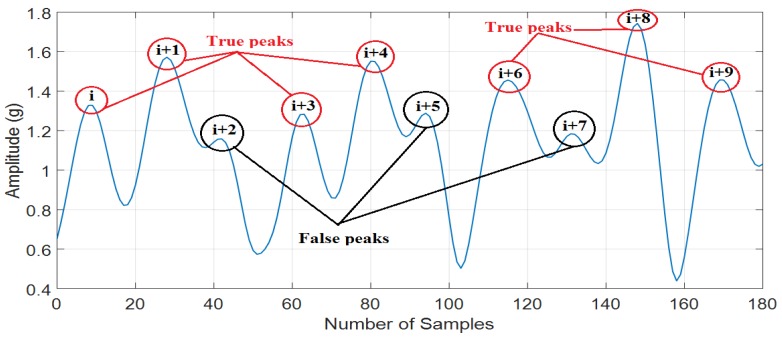
The true peaks and the false peaks of a data sample.

**Figure 7:**
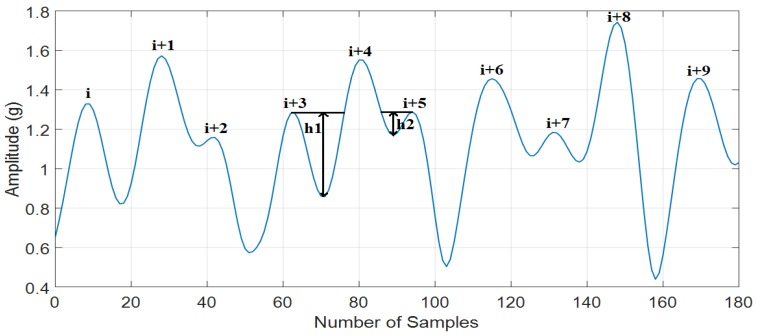
The minimal peak prominence analysis.

Based on the results in Table 1, it can be seen that the false peaks (i+2), (i+5), and (i+7) are eliminated easily by using the minimal peak prominence threshold because the minimal peak prominence of these peaks is much shorter than the true peaks (i), (i+1), (i+3), (i+4), (i+6), (i+8), and (i+9) (see Table 1). The details of the analysis process are described in the Figure 6 and Figure 7 below.

Applying minimal peak prominence can remove interferences, noises, and unexpected parts in signals that cause false step counting. By using minimal peak prominence, the peak P0 in Reference [12] can be easily eliminated. Figure 6 is an example to illustrate minimal peak prominence feature:

Using threshold can easily eliminate the false peaks (i + 2) and (i + 7). Nevertheless, it is difficult to distinguish between the true peak (i + 3) and false peak (i + 5) in Figure 6. While (i + 5) is a false peak, its height value is higher than (i + 3). Based on Figure 7, the minimal peak prominence of (i + 3) and (i + 5) is h1 and h2, respectively. The value of h2 is much lower than h1. Therefore, the false peak (i + 5) can be easily eliminated by using the minimal peak prominence feature. Hence, minimal peak prominence is a very powerful feature to eliminate false peaks.

Furthermore, the minimal peak prominence threshold will depend on the state of the person who is carrying the device. Based on the experimental results, we have proposed novel threshold values for minimal peak prominence feature, which is suitable with the state of a person to increase the performance of the system, as shown in Table 2.

● Dynamic Thresholding: The dynamic thresholding is developed to improve the limitation of a hard threshold. Dynamic thresholding is built based on the maximal and minimal values of acceleration data in a window size. The value of window size directly affects the accuracy of the system and it depends on the motion states of the user. Normally, the walking speeds and the time for each step in slow, normal, and fast walking states are shown in Table 3 [6,7].

The window size for each state will depend on the average time period for each step, as shown in formula 3:

*win*_*size* = ⎣*Ts* × *Fs*⎤ − 1, (3)

where *Ts* is the average time period for each motion state, *Ts* = 0.635 s for slow walking, *Ts* = 0.507 s for normal walking, and *Ts* = 0.469 s for fast walking; *Fs* is the sampling rate (*Fs* = 50 Hz). The average dynamic thresholding is calculated based on the maximum and minimum values of acceleration in a window size, as shown in formula 4. Then, we will compare between peak *Acc*(*j*) and the average dynamic thresholding to eliminate the false peak, as shown formula (5):

(4) $${\mathrm{Aver}}_{D}=\frac{max{\left(Acc\left(j\right)\right)}_{win\_size}+min{\left(Acc\left(j\right)\right)}_{win\_size}}{2},$$

*Th_D_* = *Acc*(*j*) − Aver_D_, (5)

where Aver_D_ is the average dynamic thresholding, *Th_D_* is the difference between the peak *Acc*(*j*) and average dynamic thresholding. The dynamic thresholding in our system is important because it can keep the correct peaks as well as to remove false peaks. Furthermore, the walking movements (e.g., strength, frequency, and habit) are different in each person. Hence, the hard threshold is not stable, it may be good for this person, but it is bad for others, such as those having large and light steps. In our proposed system, *Th_D_* is chosen to be equal to 0.15 (g).

**Figure 8:**
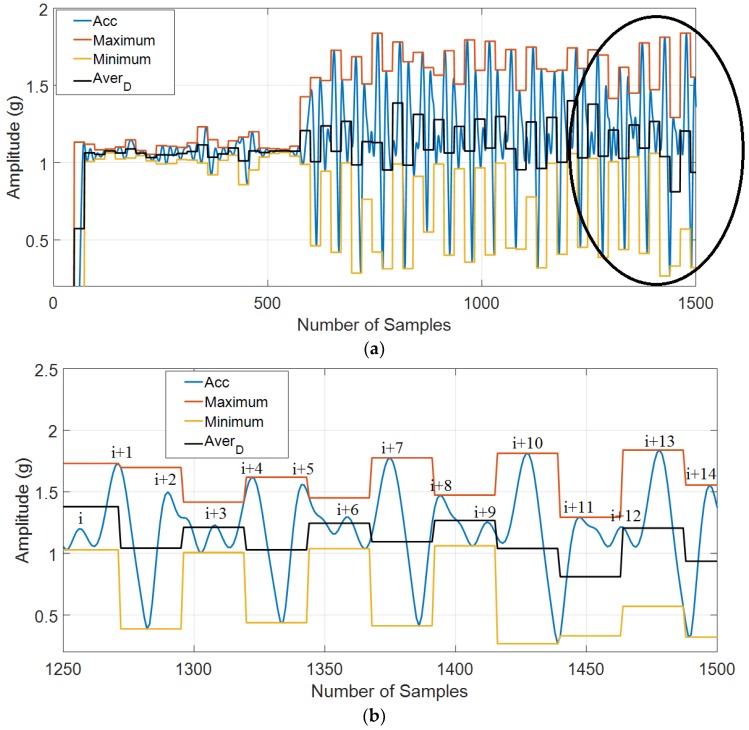
The dynamic thresholding and the zoom in dynamic thresholding for our recorded data: (**a**) The dynamic thresholding for our recorded data; and (**b**) The zoom in of dynamic thresholding for our recorded data.

From the results of Figure 8, it can be seen that the false peaks (i), (i + 3), (i + 6), (i + 9), and (i + 12) are eliminated easily by using dynamic thresholding. Furthermore, the true peaks (i + 1), (i + 2), (i + 4), (i + 5), (i + 7), (i + 8), (i + 10), (i + 11), (i + 13), and (i + 14) are kept using our proposed dynamic thresholding.

● Vibration elimination: The vibration in signal always exists because of the noise in sensor, environment and other false walking activities. It is the cause of the over-counting or true peak elimination problems. To eliminate false peaks and enhance the performance of the proposed method; we propose the feature “vibration elimination” to solve this problem. The reading values of the accelerometer in still states oscillate approximately between [0, 1, and 0] g. Hence, vibration elimination will eliminate all peaks that fluctuate around gravity acceleration (g). The formula is used to remove vibration, as shown in formula (6):

*Vib_Eli_* = *Acc*(*j*) − g, (6)

where *Vib_Eli_* is the vibration and the difference between *Acc*(*j*) and gravity acceleration g (g ≈ 9.81 m/s^2^).

Based on the experimental results, the vibration elimination thresholds were chosen, as shown in Table 4.

● Periodicity and similarity [7]: Periodicity and similarity constraints are the features that distinguish between true peak and high peak (false peak) because the periodicity feature of different users is quite similar in natural walking and true peak also has higher similarity between the two neighboring true peaks. High peaks may cause noises or abnormal activities. Hence, the periodicity and similarity features of it are lower in comparison with the true peak [7].

From the data in Table 2, it can be seen that the time for each step (slow walking, normal walking, and fast walking) is in the range from 0.45 s to 0.65 s. Periodicity and similarity features are powerful techniques to solve the false walking problem. Periodicity is a condition to check the time difference between two consecutive peaks, as shown in the formula below [7]:

(7) $$T_{i}=\frac{t_{{Peak}_{i+1}}-t_{{Peak}_{i}}}{Fs},$$

where $t_{{Peak}_{i+1}}$ and $t_{{Peak}_{i}}$ are the time of *Peak*(*i*+1) and *Peak*(*i*); *T_i_* is the time range between two neighboring *Peak*(*i*+1) and *Peak*(*i*).

Similarity is an efficient feature in noise elimination because this feature is based on the similarities between two neighboring peaks which are executed by the left or the right leg because the steps executed by the same leg are quite similar. Nevertheless, the similarity may be the cause of the under-counting problem, as the true peak will be eliminated because of false peak detection. Thus, both true and false peaks are eliminated (see Figure 11). The formula below is the condition to check the similarity feature [7]:

*S_i_* = −‖*Peak_i_*_+2_ − *Peak_i_*‖, (8)

where *Peak_i_*_+2_ and *Peak_i_* are acceleration of *Peak*(*i*+2) and *Peak*(*i*); *S_i_* is the similarity between two neighboring peaks *Peak_i_*_+2_ and *Peak_i_*.

Phase 4: Step detecting and counting: A peak confirms a correct step when it satisfies all our proposed features, periodicity and similarity features. If a peak does not satisfy any of those conditions, it will be removed. 

## 3. Results

### 3.1. The Experimental Setup

The proposed indoor positioning system integrates a low-cost 9-DOF IMU MPU-9250 (9-axis Motion Processing Unit), a wireless data transmitter/receiver, a barometer BMP180, and other supporting sensors for data fusion. 

**Figure 9:**
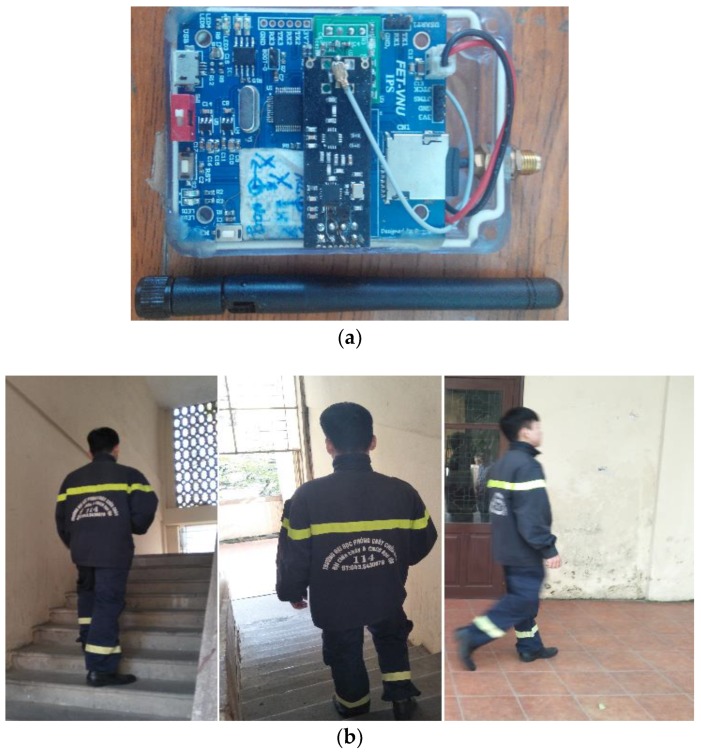
Our proposed system and a volunteer recording data: (**a**) Our proposed system; and (**b**) A volunteer is recording data.

The process of recording data was gathered from the experiment that was executed on eight male volunteers with the age: 18–28, height: 1.65–1.78 m, and weight: 58–76 kg who were selected from The University of Fire Fighting and Prevention (UFFP). The volunteers carried our system in trouser pockets and five types of data were recorded: normal walking, fast walking, slow walking, free walking, and false walking. Each type of data was recorded three times for the testing process. Then, six volunteers carried our device in their trousers’ pocket to record data for comparison with step counting based on periodicity, similarity, continuity method, and step counting based on peak detection method; two other volunteers carried our device and three comparison phones (which were installed with three popular applications: Health app on IPhone 5s, S-Health on Galaxy S5, and Pedometer and Weight Loss Coach installed on Lenovo P780) in the trouser pockets and performed 500 steps five times in free walking with the same conditions. For free walking, the volunteers walked with normal speed on the floor and up and down stairs. Figure 9 is our proposed indoor positioning system and data is being recorded with a firefighter in the free walking state.

### 3.2. Testing Process

**Figure 10:**
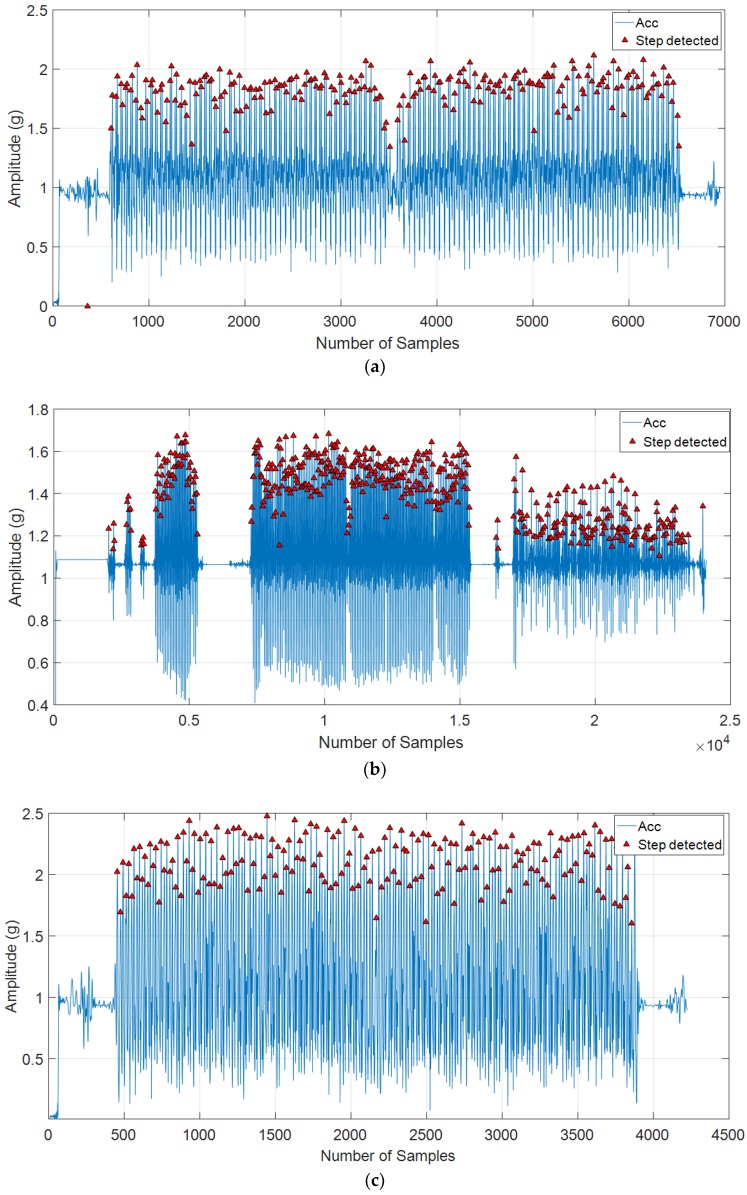

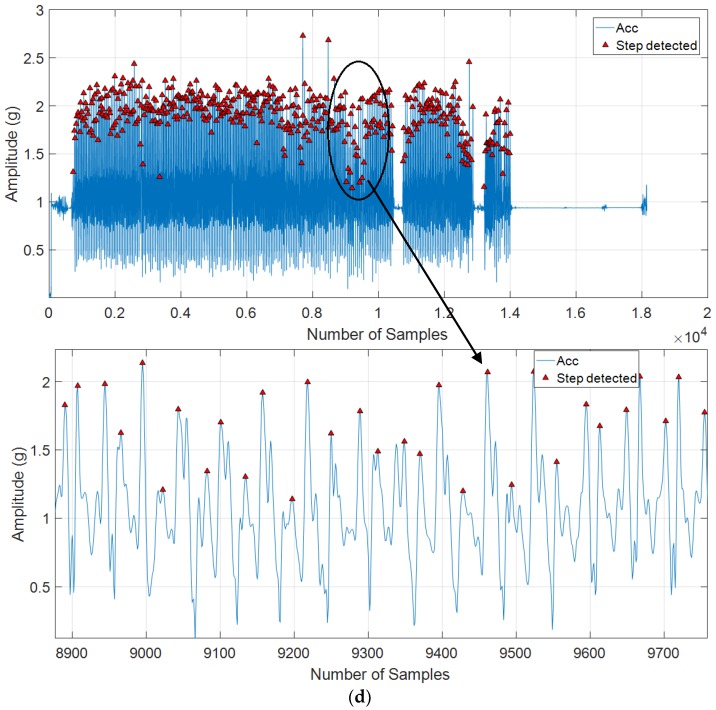
The estimated step numbers in various kinds of walking speeds: (**a**) Results after executing 227 steps of normal walking with the distance of 162 m; (**b**) Results after executing 500 steps of slow and intermittent walking; (**c**) Results after executing 187 steps of fast walking with the distance of 162 m; and (**d**) Results after executing 500 steps in free walking (floor walking, up stairs, down stairs).

The proposed method has been tested with various kinds of walking states comprising normal walking, fast walking, slow walking, and false walking. The results in Figure 10 showed that the proposed method detected the number of steps with an ultra-high accuracy: 227/227 steps detected in normal walking (see Figure 10a), 500/500 steps in slow and intermittent walking (see Figure 10b), 187/188 steps in fast walking (see Figure 10c), and 500/500 steps in free walking (see Figure 10d).

## 4. Discussion

### 4.1. Comparison with Other Methods

To evaluate the performance of our proposed step counting method, we have used the following formula to estimate the error of the proposed method [7]:

(9) $$\mathrm{Error}=\frac{\left|E-T\right|}{T}\times 100\%,$$

where E is the estimated number of steps by our method and T is the number of true steps.

**Figure 11:**
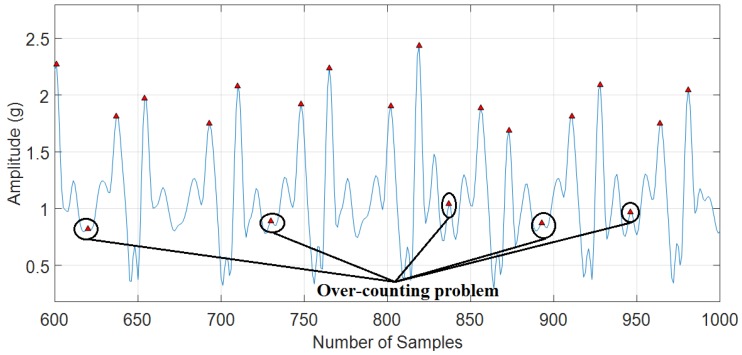
Over-counting problem in step counting based peak detection method [7].

**Figure 12:**
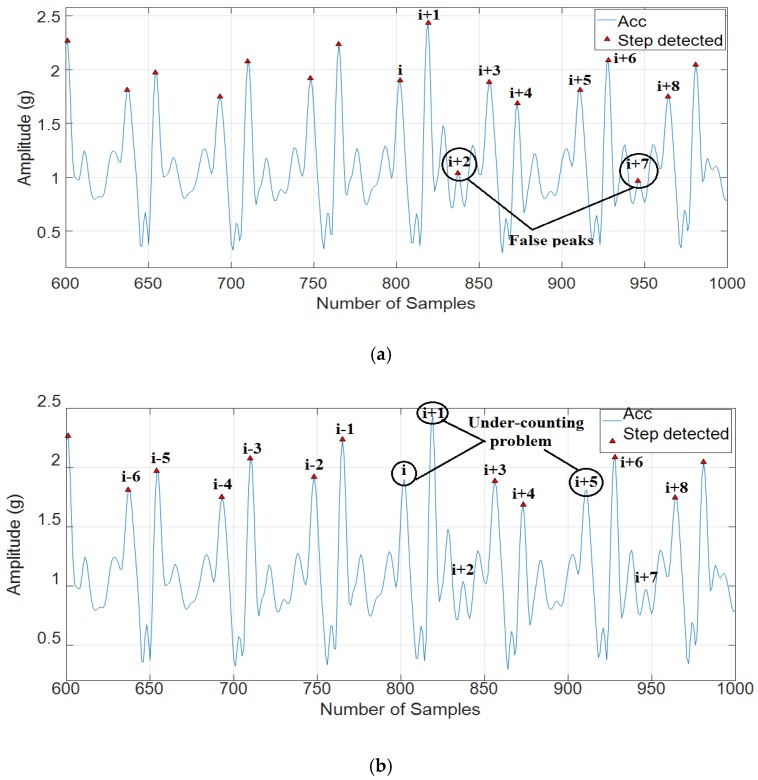
Analyze step counting based on periodicity, similarity, and continuity method: (**a**) False peaks detected in step counting after using periodicity and continuity features; and (**b**) Under-counting problem after using similarity feature.

In this paper, we have compared our proposed method with two published methods consisting of step counting based on periodicity, similarity, and continuity [7] and step counting based on a peak detection method in four types of activities: Fast walking, normal walking, slow walking, and false walking. Based on experimental results, it can be seen that step counting, based on the peak detection method, is still subject to over-counting problem (see Figure 11 for more detail), although we chose a suitable minimal peak distance. Besides, step counting based on periodicity, similarity, and continuity can effectively solve the false walking problem, but this method also reveals limitations in counting the number of steps in true walking because of the noise in low-cost IMU. Low-cost IMU is subject to noises which are the cause of the true peak elimination problem when using the similarity feature to recognize steps. For example, the true peaks (i), (i+1), and (i+5) were eliminated in step counting based on periodicity, similarity, and continuity (sees Figure 12b for more detail).

The similarities between two neighboring left or right steps such as the similarities between (i − 6), (i − 4), (i − 2), (i), (i + 3), (i + 5), and (i + 8) in the left steps, or (i − 5), (i − 3), (i − 1), (i + 1), (i + 4), and (i + 6) in the right steps in Figure 12b are quite close, while the similarity in the false walking is unobvious. Based on Figure 12a, the peaks (i + 2) and (i + 7) are false peaks because of noise in low-cost IMU. It will be cause of the under-counting problem in Figure 12b, the true peaks (i), (i + 1), and (i+5) were eliminated because of false peaks (i + 2) and (i + 7) being detected. The similarity is a very good feature to recognize true steps. Nevertheless, it will eliminate both true peak and false peak in noisy signals. Hence, the performance of step counting, based on periodicity, similarity, and continuity method is unstable in noisy signals.

**Figure 13:**
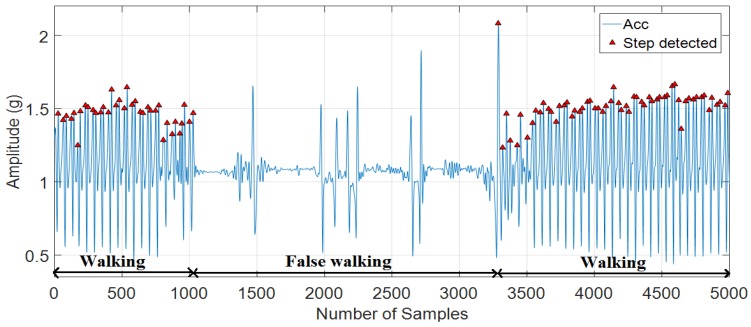
Our proposed algorithm can eliminate false walking while keeping step counting in normal walking.

Based on the limitations of current researches, four features have been proposed in our method to eliminate all false peaks of noisy signals. Our proposed features combined periodicity and similarity features to solve the false walking problem. Figure 13 is the result of our proposed method. It can be seen that all false peaks are eliminated, while all true peaks are kept.

**Figure 14:**
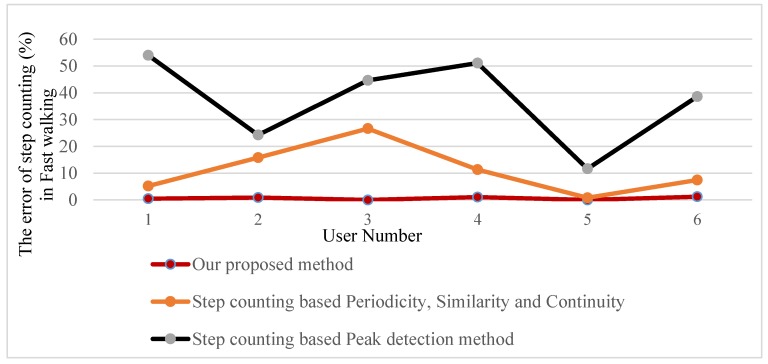

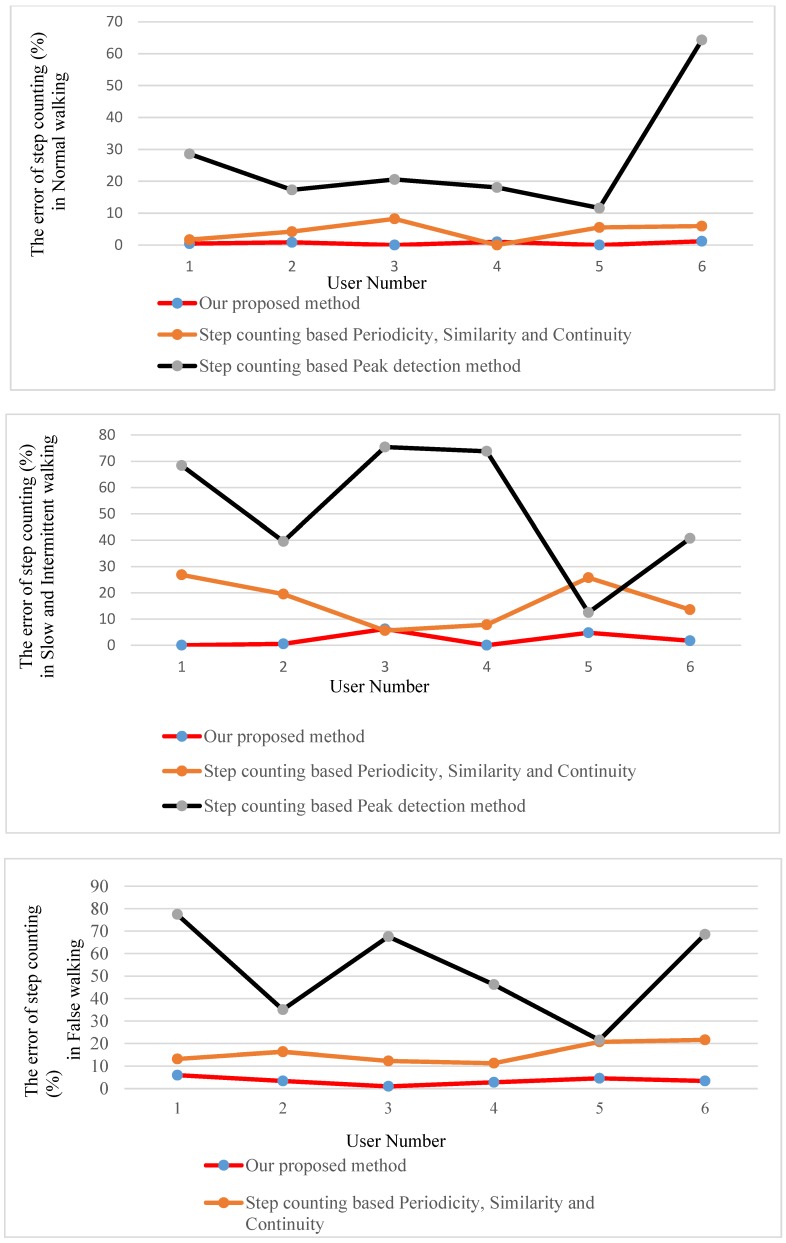
The comparison among our proposed method and two methods presented in Reference [7].

Figure 14 shows the comparisons among our proposed method; step counting based on periodicity, similarity, continuity method, and step counting based on the peak detection method in fast walking, normal walking, slow walking, and false walking.

The results in Table 5 shows that our proposed method has ultra-high improvement in comparison with step counting based periodicity, similarity, continuity method, and peak detection based method in all states. For free walking, the average error of our proposed method is only 0.58%; the figures for step counting based periodicity, similarity, continuity method, and peak detection method are 11.18% and 37.35%, respectively. Our proposed method also effectively solves false walking problems with an average error of only 3.53%, while the average error for step counting based on periodicity, similarity, continuity method, and the peak detection based methods are 15.90% and 52.70%, respectively.

Our proposed method can detect exactly the number of steps in both true and false walking at various speeds, achieving highly accurate, and stable performance in both true walking and false walking, as shown in Table 5, as we have proposed the suitable cutoff frequency in a low-pass filter and the proposed features are adaptive to the states of the user. Furthermore, minimal peak prominence and dynamic thresholding are novel and powerful features that can eliminate most of the false peaks. Hence, our proposed method solves under-counting, over-counting, and false walking problems better than other methods in publications.

### 4.2. Comparison with Other Commercial Applications

To compare with other commercial applications, two volunteers carried our device and three comparison phones in their trouser pockets and executed the experiment at the same time and conditions. 

From the results shown in Table 6, it can be seen that the Pedometer and Weight Loss Coach installed on Lenovo P780 has the worst performance, even though it has had more than 10 million downloads on Google Play [30] (accessed on 10 June 2018). Furthermore, this app also exhibits over-counting while the person is riding a motor cycle or driving a car with the phone in his trouser pocket. The i-Health app on IPhone 5s (IOs 10.3.3) does not count steps while driving a motor or car. Nevertheless, in the same conditions of testing, this app achieved lower accuracy in comparison with our proposed method and S-Heath on Galaxy S5 (Android 6.01). Furthermore, i-Health works without real-time response and it cannot count steps when we combine walking and controlling a motor/car. Our proposed method and S-Health have approximately the same average error results of 0.16% and 0.32%, respectively. Moreover, our proposed method can solve the over-counting problem in the false waking state, e.g., controlling a motor cycle or car, sitting in a moving car, and swinging. The analysis shows that the reliability of a step counting method depends on both algorithm and type of sensor. We proposed the use low-cost IMU to reduce the cost of the system. After carefully calibrating to eliminate the noise, our proposed method can reliably and exactly detect the number of steps with promising results in comparison with three popular commercial applications in the same conditions of testing (see Table 6).

The proposed method has resulted in significant improvement in the accuracy for both true and false walking in comparison with the previous step counting methods and several popular applications. Furthermore, it provides real-time response.

### 4.3. Testing with Public Datasets

To improve the ability of the proposed algorithm, we tested our proposed algorithm on public datasets [34] with the same parameters. Data of the public datasets were collected by Samsung S6 in different positions: Hand, frontpocket, backpocket, neck pouch, bag, and armband. The public datasets [34] are available at: https://github.com/Oxford-step-counter/DataSet/tree/master/validation (Accessed on 30 August 2018). The proposed algorithm also achieved very good results of 97.04% for the average of accuracy, the detail results are as Table 7 below:

The achieved accuracy on public datasets is insignificantly lower than that recorded from ours. The step counting results by our proposed algorithm on public datasets were added in the source: https://github.com/Pham-Van-Thanh/Step-counting.

## 5. Conclusions

In this paper, we have successfully developed a highly accurate step counting with real-time response using a low-cost 3-DoF accelerometer for various walking states. We have successfully solved the over-counting, under-counting, and false walking problems by proposing four features: Minimal peak distance, minimal peak prominence, dynamic thresholding, and vibration elimination in combination with peak detection and periodicity and similarity features. Minimal peak prominence and dynamic thresholding are novel and powerful techniques in false peak elimination in step counting. In the future, we will expand to detect steps in a crawling state to support indoor positioning systems to predict the positions of on-duty firefighters.

We have been published two recorded data and simulation results of our proposed step counting method on github.com for checking and comparing.

The source: https://github.com/Pham-Van-Thanh/Step-counting. 

## Figures and Tables

**Table 1:** The minimal peak prominence analysis.

The Peak	Horizontal Line Extends to the Left and	Horizontal Line Extends to the Right and	Lowest Point on the Left Interval	Lowest Point on the Right Interval	Minimal Peak Prominence of the Peak (the Difference between the Peak and)
(i)	Reaches the left end	Crosses the peak (i+1)	Left endpoint	a	a
(i+1)	Reaches the left end	Crosses the peak (i+8)	Left endpoint	f	Left endpoint
(i+2)	Crosses the peak (i+1)	Crosses the peak (i+3)	b	c	b
(i+3)	Crosses the peak (i+1)	Crosses the peak (i+4)	c	d	d
(i+4)	Crosses the peak (i+1)	Crosses the peak (i+8)	c	f	c
(i+5)	Crosses the peak (i+4)	Crosses the peak (i+6)	e	f	e
(i+6)	Crosses the peak (i+4)	Crosses the peak (i+8)	f	h	h
(i+7)	Crosses the peak (i+6)	Crosses the peak (i+8)	g	h	g
(i+8)	Reaches the left end	Reaches the right end	f	k	f
(i+9)	Crosses the peak (i+8)	Reaches the right end	k	l	l

**Table 2:** The minimal peak prominence threshold for fast walking, normal walking, and slow walking states.

The States	Minimal Peak Prominence Threshold (H)
Fast walking	0.35 g
Normal walking	0.25 g
Slow walking	0.2 g

**Table 3:** Speed information, average speed, step length and the average time period for each step Ts(s).

Speed Level		Average Speed and Step Length for Each State	The Average Time Period for Each Step Ranges Ts(s)
Slow walking	Mean ± Std (m/s) Step length (m)	0.937 ± 0.040 (m/s) 0.595 (m)	0.635 (s)
Normal walking	Mean ± Std (m/s) Step length (m)	1.360 ± 0.037 (m/s) 0.690 (m)	0.507 (s)
Fast walking	Mean ± Std (m/s) Step length (m)	1.70 ± 0.065 (m/s) 0.797 (m)	0.469 (s)

**Table 4:** The vibration elimination threshold for fast walking, normal walking, and slow walking states.

The States	*Th_Vib_*^1^ Threshold
Fast walking	0.15 g
Normal walking	0.1 g
Slow walking	0.08 g

^1^*Th_Vib_* is vibration elimination threshold.

**Table 5:** The average errors among our proposed method and two methods presented in Reference [7].

The States	Our Propose Method	Step Counting Based Periodicity, Similarity and Continuity [7]	Step Counting Based Peak Detection Method [7]
Free walking	0.58%	11.18%	37.35%
Fast walking	1.06%	4.26%	26.73%
Slow walking	2.42%	14.92%	96.77%
False walking	3.53%	15.90%	52.70%

**Table 6:** Comparisons between our proposed system with different commercial applications on 500 steps of free walking.

Times	1	2	3	4	5	Average Number of Steps	Average Error
True steps	500	500	500	500	500	500	0
Our proposed system	500	498	499	500	499	499.2	0.16%
S-Health on Galaxy S5 (Android 6.01) [31]	500	502	498	503	499	500.4	0.32%
Health App on IPhone 5s (iOS 10.3.3) [32]	499	507	489	477	482	490.8	2.40%
Pedometer & Weight Loss Coach installed on Lenovo P780 [33]	461	448	376	483	472	448	10.40%

**Table 7:** Performance with Public Datasets

User No.	Positions	True Steps	Our Proposed Algorithm	Accuracy
1	Hand	326	323	99.08%
	Front_pocket	327	325	99.39%
	Back_pocket	343	327	95.34%
	Neck pouch	346	339	97.98%
	Bag	346	333	96.24%
	Armband	335	335	100%
2	Hand	340	332	97.65%
	Front_pocket	343	329	95.92%
	Back_pocket	337	335	99.41%
	Neck pouch	360	348	96.67%
	Bag	361	322	89.2%
	Armband	343	335	97.67%
**Average accuracy**				**97.04%**
